# Gene coexpression networks reveal molecular interactions underlying cichlid jaw modularity

**DOI:** 10.1186/s12862-021-01787-9

**Published:** 2021-04-22

**Authors:** Pooja Singh, Ehsan Pashay Ahi, Christian Sturmbauer

**Affiliations:** 1 Institute of Biology, University of Graz, Universitätsplatz 2, 8010 Graz, Austria; 2 Department of Biological Sciences, University of Calgary, 2500 University Dr NW, Calgary, AB Canada; 3 Organismal and Evolutionary Biology Research Programme, University of Helsinki, Viikinkaari 9, 00014 Helsinki, Finland

**Keywords:** Modularity, Coexpression network, Jaw morphogenesis, Trophic adaptation

## Abstract

**Background:**

The oral and pharyngeal jaw of cichlid fishes are a classic example of evolutionary modularity as their functional decoupling boosted trophic diversification and contributed to the success of cichlid adaptive radiations. Most studies until now have focused on the functional, morphological, or genetic aspects of cichlid jaw modularity. Here we extend this concept to include transcriptional modularity by sequencing whole transcriptomes of the two jaws and comparing their gene coexpression networks.

**Results:**

We show that transcriptional decoupling of gene expression underlies the functional decoupling of cichlid oral and pharyngeal jaw apparatus and the two units are evolving independently in recently diverged cichlid species from Lake Tanganyika. Oral and pharyngeal jaw coexpression networks reflect the common origin of the jaw regulatory program as there is high preservation of gene coexpression modules between the two sets of jaws. However, there is substantial rewiring of genetic architecture within those modules. We define a global jaw coexpression network and highlight jaw-specific and species-specific modules within it. Furthermore, we annotate a comprehensive in silico gene regulatory network linking the Wnt and AHR signalling pathways to jaw morphogenesis and response to environmental cues, respectively. Components of these pathways are significantly differentially expressed between the oral and pharyngeal jaw apparatus.

**Conclusion:**

This study describes the concerted expression of many genes in cichlid oral and pharyngeal jaw apparatus at the onset of the independent life of cichlid fishes. Our findings suggest that – on the basis of an ancestral gill arch network—transcriptional rewiring may have driven the modular evolution of the oral and pharyngeal jaws, highlighting the evolutionary significance of gene network reuse. The gene coexpression and in silico regulatory networks presented here are intended as resource for future studies on the genetics of vertebrate jaw morphogenesis and trophic adaptation.

**Supplementary Information:**

The online version contains supplementary material available at 10.1186/s12862-021-01787-9.

## Background

Evolutionary modularity, or the degree to which traits form higher-order units that evolve independently, has emerged as a central concept in evolutionary biology, especially in the last two decades [1–8]. It is now viewed as a major mechanism governing evolvability (i.e. potential for adaptive change). Units that exhibit strong within-module integration but relatively weak between-module integration are considered modular and there can be various degrees of modularity governing complex phenotypes [6]. Heterogeneity in integration enables adaptive flexibility, with low degrees of integration being associated with high phenotypic change [9]. Modularity is considered a major driver of phenotypic evolution [10, 11] and operates at many biological levels such as morphology, function, development, and genetic architecture (reviewed in [6]). Thus, modularity can also be observed at the molecular level, for example in gene regulatory networks [12]. Transcriptional modularity can bring about complex developmental changes and give biological systems greater ability to respond to changes with minimal interference with genomic complexity [13–15].

The vertebrate craniofacial anatomy is amongst the most complex musculoskeletal systems with highly modular compartmentalisation, representing a key innovation of the Craniota. Cichlid fishes are particularly diverse in terms of their craniofacial anatomy and are renowned for having two sets of jaws (oral and pharyngeal) that are a hallmark example of modularity [16]. The functional decoupling of these jaws is seen as a key innovation that permitted the oral jaw apparatus (OJA) to be solely dedicated to prey capture and the pharyngeal jaw apparatus (PJA) to prey processing, allowing for independent evolution. Their adaptive radiation in several East African lakes was hypothesised to be connected to their great efficiency to adapt to novel food sources [17]. Another factor facilitating adaptive radiation is phenotypic plasticity of the jaw apparatus that enabled rapid response to environmental changes [18]. Remarkably, similar craniofacial morphologies evolved in parallel across the radiations of the three Great East African Lakes (Lake Tanganyika, Malawi and Victoria) in response to similar selection regimes. The modularity of cichlid jaws has been an active area of research [19–22] as it can shed light not only on how cichlid jaws adapted to different diet regimes, but also to understand the role of modularity in facilitating diversification, even repeatedly [23]. The theory of morphological integration postulates that functionally modular traits will be inherited independently, and thus be genetically modular too [1]. So far, studies have provided evidence of cichlids jaws being not only functionally and mechanistically modular [19, 24] but also modular at the genetic level [16, 21, 25]. There is also mounting evidence of these two major units displaying high within-module integration [16, 26]. However, modularity at the transcriptional level in global gene expression has not been yet been investigated in detail.

Gene regulatory networks (GRNs) govern development in multicellular organisms. Modules (i.e. sub-circuits) within GRNs allow for parts of the network to evolve, without interfering with the rest of the network. Within each module, gene associations represent regulatory links that are mainly determined by presence of common transcription factor binding sites in their regulatory sequences [27]. Alterations of these links in the ancestral source structure, especially via *cis*-regulatory changes, can bring about diverse developmental and evolutionary change in derived structures [27]. The reuse and re-wiring of GRNs plays a major role in morphological evolution [28] and there are impressive examples in insects and crustaceans of GRN rewiring with varying levels of sub-circuit conservation (reviewed in [27]). In cichlids, it was previously shown that an ancient gene network was redeployed to give rise to oral and pharyngeal teeth [29]. However, it is not known if this also was the case for the two jaws.

During development and morphogenesis of the jaw musculoskeletal apparatus, a high number of highly interconnected molecular cascades (e.g. Wnt, BMP, Hedgehog, Notch, retinoic acid, growth factors and calcium dependent pathways) mediate their signals to regulate overlapping GRNs (reviewed in [30]). In cichlids, as one of the most diverse groups of vertebrates in jaw morphology, elucidating the related GRNs is an important step to understand the molecular basis of their functional modularity, phenotypic plasticity, and the evolution of parallel eco-morphologies. To date, the GRNs underlying cichlid jaw development are largely unknown, with only one attempt thus far using a set of candidate genes to described lower pharyngeal jaw plasticity at the GRN level [31].

By using data from whole transcriptome sequencing, it is possible to build gene coexpression networks (GCNs) and study the degrees of transcriptional connectivity at a broad scale [32]. GCNs are biologically interesting as they reveal clusters of genes that are expressed in symphony and thus may be under the control of the same transcriptional regulatory program [33]. Functionally annotating GCNs can shed light onto GRN architecture. Nodes in GCNs are represented by genes and highly interconnected (coexpressed) nodes are called modules. Genes that are coexpressed are considered to be co-regulated and be constituents of the same pathway or have the same upstream regulator(s), and thus be functionally related [34]. The direction of regulation cannot be deduced from GCNs however. This approach has been applied extensively in model organisms and recently successfully been applied to study key drivers of speciation and ecological phenotypes [35–39] and seems promising to identify the GCNs driving the functional modularity of cichlid oral and pharyngeal jaws.

Here we set out to delineate the gene coexpression networks of cichlid oral versus pharyngeal jaws and yield insight into the regulatory mechanisms underlying their evolutionary modularity. To address this, we sequenced whole transcriptomes of OJA and lower PJA (LPJA) from three cichlid species (*Gnathochromis pfefferi*, *Ctenochromis horei*, and *Limnotilapia dardennii*) with different prey spectres, prey capture strategies and distinct ecomorphologies. These species comprise a monophyletic and recently diverged clade (~ 3.56 MYA) [P. Singh, unpublished observations] from the tribe Tropheini from Lake Tanganyika, that are named after their impressive trophic diversity. *G. pfefferi* has a longer snout that is specialised for picking invertebrates such as shrimp/insects from the soft lake bottom [40]. *C. horei* is the most specialised fry-eater in the Tropheini with a terminal mouth and *L. dardennii* is an omnivore [57]. We used weighted gene coexpression network analysis (WGCNA) to identify major coexpression modules that may play a role in shaping their evolutionary and functional disparities with regards to skeletogenesis and environmental cues (i.e. plasticity). The monophyly and relatively recent coancestry of these species is advantageous as re-wiring of ancestral gene coexpression networks potentially driving oral and pharyngeal jaw adaptations will be easier to distinguish from background noise. Assuming some degree of conservation, we transferred the knowledge of functionally validated annotations from literature on model organisms to in silico annotated GRNs in cichlid jaws. We specifically focused on an ecologically informative developmental stage (stage 26, [41]) where post-embryonic development of both jaws is complete and the larvae are ready to feed independently and interact with the environment. We have previously shown that at this stage the jaws of species appear to be morphologically distinct [42]. Our new results provide insight into the transcriptional interactions that shaped oral and pharyngeal modularity in Lake Tanganyika cichlids.

## Results

### Gene expression patterns of oral and pharyngeal jaws

**Fig. 1:**
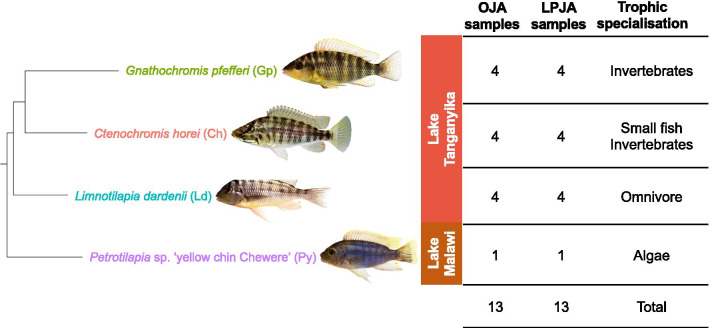
Study design. The lake, trophic niche, phylogenetic relationships and number of cichlid species sequenced in this study. Fish photographs: W. Gessl (University of Graz). Phylogeny adapted from Irisarri*, Singh* et al. 2018

**Fig. 2:**
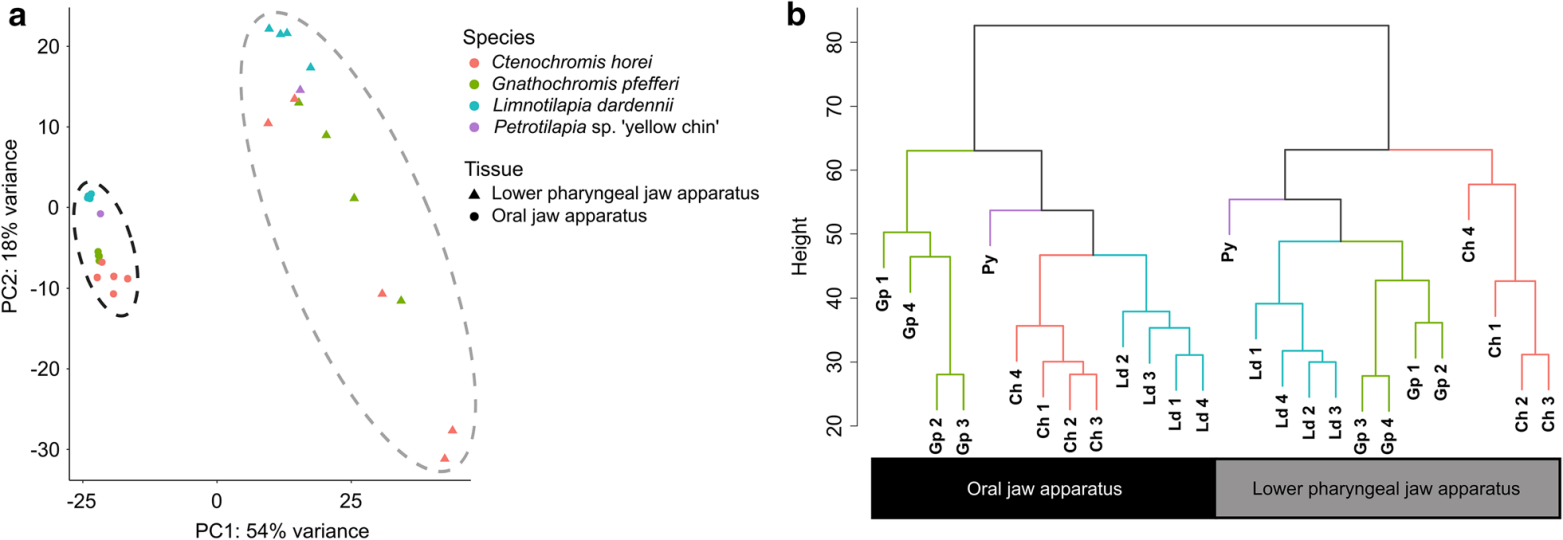
**a** Principal component analysis (PCA) and **b** hierarchical clustering dendrogram of normalised expression counts of 16,669 *O. niloticus* annotated genes in 26 RNA-seq samples from the oral jaw apparatus (OJA) and lower pharyngeal jaw apparatus (LPJA). *Ctenochromis horei* (Ch), *Gnathochromis pfefferi* (Gp), *Limnotilapia dardennii* (Ld) and *Petrotilapia* sp. ‘yellow chin Chewere’ (Py)

Approximately 11 million paired-end reads per sample (125 bp in length) were obtained from Illumina Hi-Sequencing Technology for four biological replicates per jaw per Lake Tanganyika species and one biological replicate per jaw for the Lake Malawi species (Table 1). After filtering, approximately 63% of the reads per sample mapped to the reference genome as proper pairs (Additional file 2: File S1). To identify the relationship between the transcriptomes of the jaws and species in this study (Fig. 1), we extracted the expression of 16,669 genes from each sample and conducted hierarchical clustering analysis based on Euclidean distance. A clear pattern emerged in the clustering dendrogram (Fig. 2). Gene expression of the samples showed jaw-specific clustering in both the PCA (Fig. 2a) and dendrogram (Fig. 2b), irrespective of trophic niche or lake of origin, indicating that the OJA and LPJA have highly conserved gene expression signatures. The gene expression of the LPJA suggests higher variance on both PC1 and PC2 compared to the OJA, even within the same species. Within the jaw-specific clusters in the dendrogram, the samples were clustered according to species. However, the species clustering of OJA samples was different from LPJA samples and neither reflected the phylogenetic relationship of the species shown in (Fig. 1 phylogeny based on Irisarri*, Singh* et al. 2018). In the OJA samples, *C. horei* and *L. dardennii* were closer to each other than the *G. pfefferi* but in the LPJA samples, *G. pfefferi* and *L. dardennii* were closer to each other than the *C. horei*. Notably, the Lake Malawi species, *Petrotilapia* sp. ‘yellow chin Chewere’, was embedded within the Lake Tanganyika samples, but held different positions within the OJA compared to LPJA. *Petrotilapia* sp. ‘yellow chin Chewere’ is a sister lineage to the Lake Tanganyika Tropheini (Fig. 1) and since we only one had one biological replicate per jaw for it, it was not included in the subsequent co-expression network and differential gene expression analyses.

**Table 1:** Glossary of coexpression network terms

Term	Description
Modularity	Modularity is the innate property of networks, it has been studied across different scientific disciplines. In this manuscript, we use it only to refer to the functional modularity between cichlid oral and pharyngeal jaws, unless otherwise specified
Module	A cluster of highly interconnected nodes
Nodes	Denote genes
Edges	Denote the connections between genes (nodes). In a weighted network, edges are attributed a “weight” based on the Pearson correlation of gene expression between two genes
Connectivity	In a weighted network, connectivity of a node is calculated as the sum of the weight of its edges. Thus, it represents how correlated a node is with other nodes in the network
Adjacency matrix	It is a measure of gene coexpression calculated by the absolute value of the Pearson correlation coefficient of gene expression between genes, raised to a power so the degree distribution fits a small-world network
Topological overlap matrix (TOM)	Clusters the adjacency matrix with the average linkage hierarchical clustering to incorporate network topological similarity
Module Eigengene (ME)	Represents the gene expression profile of a module and is defined as the first principal component of a module
Zsummary	Is a composite statistic based on a permutation test that takes into account the connectivity and density of genes in a module
Gene significance (GS)	Gene significance is used to incorporate external information into the coexpression network. It is the Pearson correlation between genes in a module and traits of interest (i.e. jaw type or species)

### Differential gene expression between oral and pharyngeal jaws

There were 9475 genes (FDR adjusted *p-value* < 0.05) differentially expressed genes between OJA and PJA (Additional file 2: File S1). Of these 4659 were upregulated in the OJA and 4816 were upregulated in the PJA (Additional file 2: File S1). The gene enrichment analysis for the differentially expressed genes resulted in 353 significant GOs for biological processes (FDR cut-off < 0.01, Additional file 3: File S2a). In particular, GOs related to muscle and skeletal system development and morphogenesis, as well as biosynthesis of organic compounds and response to stimulus were among the most abundantly enriched biological processes (Additional file 3: File S2a). To narrow down potential gene networks determining oral versus pharyngeal jaw apparatus, we only focused on GOs related to muscle and skeletal system development and morphogenesis. In addition, we also focused on GOs related to response to organic compounds (e.g., exogenous chemicals and nutritional compounds) which could potentially provide molecular links between mechanisms involved in jaw formation and activity of environmentally responsive signals exactly when feeding starts. We retrieved the genes in GOs related to each of these biological processes and grouped them together for next analysis steps (Additional file 3: File S2a).

Of the 225 genes enriched for skeletal GO terms, of note were ones from the Wnt, BMP, hox pathways and chondrocyte genes (*col6a2, col6a1, colq, col9a2, col1a2, col11a1b, col8a1b, col28a2a*) and *dlx4b*, dlx1a, sox10 genes (Additional file 3: File S2a). We also found predicted transcription factors of Wnt pathway to be enriched in the differentially expressed genes advocating the pivotal role of Wnt in OJA versus LPJA skeletal specification. Myomesin (*myo1a, myo2a, myo18aa, myorg*) and myosin (*mybpha, mybphb, mybpc3*) genes, the myogenic regulatory factor *myf6*; *tbx20, tbx1*; and troponin genes (*tnnc1a, tnnc1b, tnnc2, tnni2a, tnnt2c, tnni3k*) were notable genes out of the 105 genes enriched in the GOs related to muscle formation processes. The transcription factor (TF) predictions also found an enrichment for the TFs of these genes/pathways in the differentially expressed genes (Additional file 3:File S2a). The greatest number of genes (434) were associated with biogenesis of organic compounds and response to stimulus GO biological processes (Additional file 3: File S2a). Several components of the Ahr pathway, but not the pathway itself, were enriched. The TF prediction for these genes found 326 TF genes, among which 235 TFs displayed differential expression between the OJA and LPJA (Additional file 2: File S1, Additional file 3:File S2a). Many of these differentially expressed TFs also appeared to be involved in musculoskeletal formation.

### Conditional gene coexpression networks

**Fig. 3:**
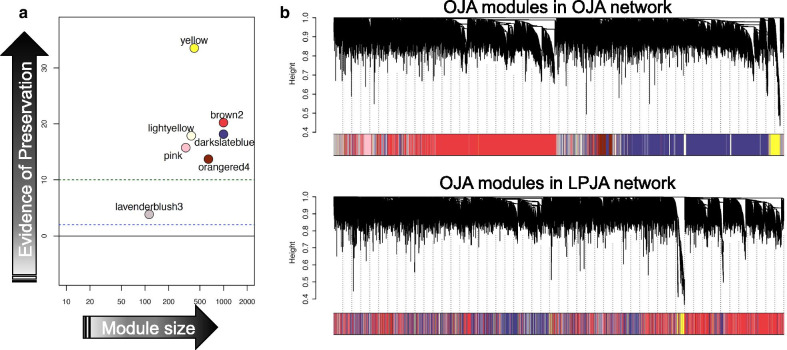
Conditional coexpression analysis: Preservation of modules in the GCNs underlying oral (OJA) and lower pharyngeal jaw apparatus (LPJA). **a** The colours represent identified OJA coexpression modules. Preservation of genes found in OJA modules in the LPJA coexpression network was calculated by a Z_summary_ statistic (y-axis) based on a permutation test that takes into account the connectivity and density of genes in a module. Z_summary_ < 2 represents lack of preservation (dotted blue line). Z_summary_ between 2 and 10 implies moderate preservation. Z_summary_ > 10 supports strong preservation of module. **b** Visual representation of module preservation. The upper panels of the dendrogram represents average linkage clustering tree based on topological overlap distance in gene expression profile. Each ‘leaf’ of the dendrogram corresponds to one gene. The lower panels of the dendrograms represent colours that correspond to OJA modules Top: OJA modules in the OJA GCN. Bottom: OJA modules in the LPJA GCN

To explore the preservation of GCN modules between cichlid OJA and LPJA transcriptomes, we first defined the GCN in one condition (i.e. OJA) and then verified the preservation of its modules in the second condition (i.e. LPJA), and vice versa. We analysed data of the OJA and LPJA samples separately and assessed module preservation between them using a composite Z_summary_ statistic. We identified seven OJA modules: yellow, brown2, lightyellow, darkslateblue, pink, orangered4 and lavenderblus3 (Fig. 3). Six of these seven modules showed strong preservation (i.e. many genes are shared between the modules of the two jaws) in the LPJA-GCN and one module showed moderate preservation (Fig. 3a). However, visualisation of the OJA modules in the LPJA-GCN showed that there was extensive re-wiring of genes of the OJA modules in the LPJ GCN (i.e. hierarchical clustering suggests the genes to be differently clustered between the two jaws) (Fig. 3b). A similar pattern was independently observed for the eight modules identified in LPJA-GCN and their preservation in the OJA-GCN (Additional file 1: Figure S4). Four LPJA modules were strongly preserved in OJA-GCN, two were moderately preserved, and two modules were not preserved. Overall, more OJA modules were preserved in the LPJA network than LPJA modules in the OJA network.

### Global jaw coexpression network

**Fig. 4:**
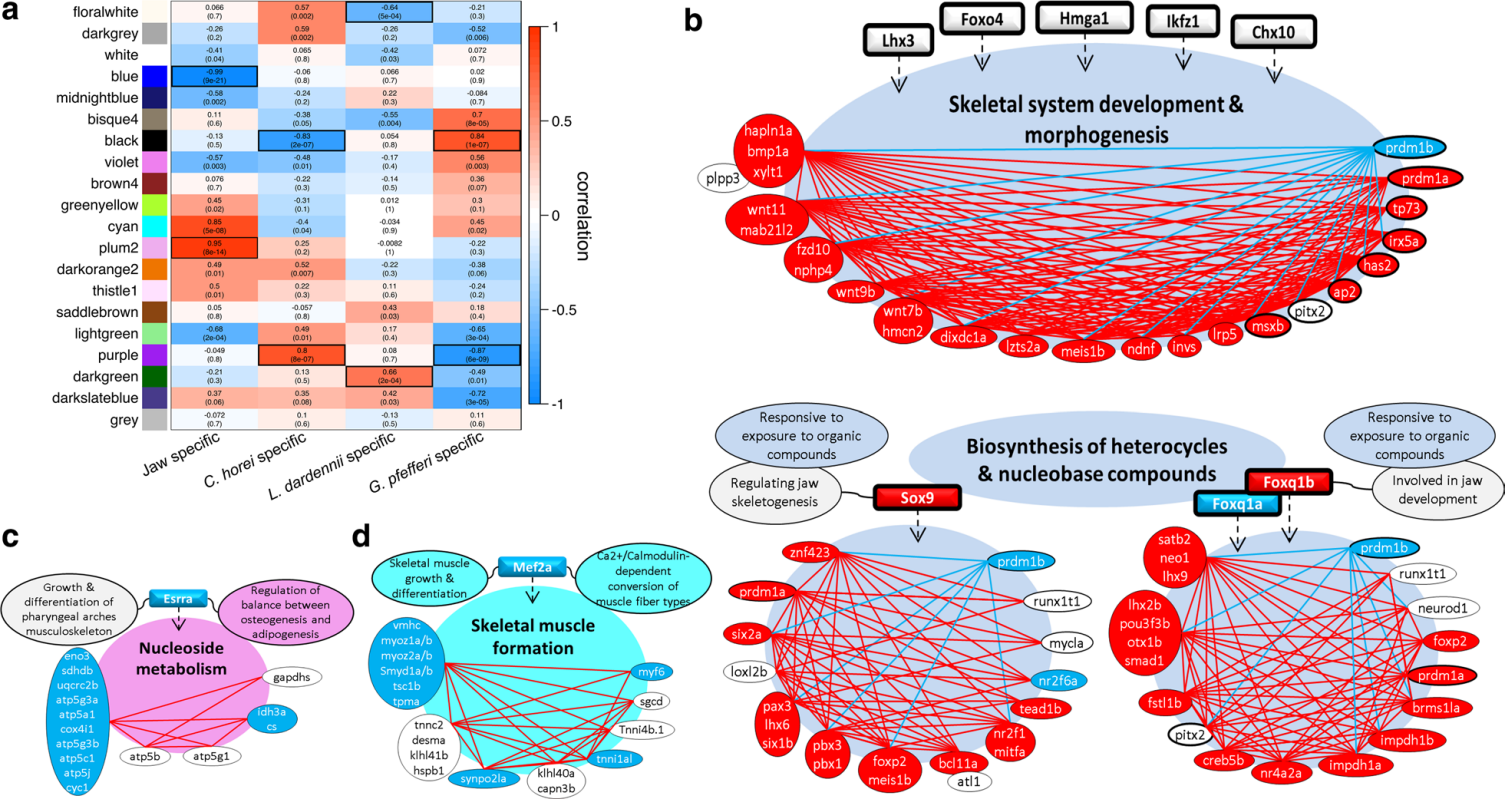
Global coexpression analysis: Coexpression sub-modules with jaw or species specific expression patterns. **a** The colours represent the coexpression modules identified using data from bots jaws. Correlation (r) score and *p*-value significance of module to jaw type or species. Red denoted strong postive correlation and blue denotes strong negative correlation. Sub-modules of coexpressed genes within **b** Blue module **c** Plum2 and **d** Cyan modules. The red and blue connecting lines between the genes in each sub-modules indicate significant positive and negative expression correlations, respectively (*P* < 0.05). The predicted TF(s) at upstream of each each sub-module are depicted in square shape gene symbol with their related functions. The red, blue and white shadings for genes respectively indicate increased expression in oral and pharyngeal jaws, and no expression difference between the jaws

To construct a global GCN of cichlid jaw apparatus we conducted the analysis using both OJA and LPJA expression data as input for WGCNA. The signed hybrid network identified 19 gene coexpression modules, and genes that did not fit into any module were placed in the “grey” module (Fig. 4a, Additional file 1: Figure S5). The 19 modules ranged in size from 35 to 1992 genes). We correlated module eigengenes with jaw apparatus type (oral or lower pharyngeal) and species to identify jaw-specific and species-specific modules (Fig. 4a). The strength of correlation was measured by the gene significance and connectivity of genes in each module (Additional file 1: Figures S6, S7). Three modules were highly correlated (|r|> = 0.8) to jaw type were: Blue (r = -0.99, *p-value* = 9e-21), Cyan (r = 0.85, *p-value* = 5e-08) and Plum2 (r = 0.95, *p-value* = 8e-14). The two highest correlated *C. horei* specific modules were black and purple (|r|> = 0.8, *p*-value < 1e-6). The two highest correlated *L. dardennii* specific modules were floralwhite and darkgreen (|r|> = 0.64, *p*-value < 1e-3). The two highest correlated *G. pfefferi* specific modules were black and purple (|r|> = 0.8, *p*-value < 1e-5). Thus, the black and purple module was shared by the two carnivores *G. pfefferi* and *C. horei*. The black module was positively correlated with *G. pfefferi* and negatively correlated with *C. horei*, while the purple module was positively correlated with *C. horei* and negatively correlated with *G. pfefferi*. These two modules were not correlated with *L. dardennii* or jaw type.

### GO annotation of jaw-specific modules

Three coexpression gene modules displaying significant expression association in a jaw-specific manner were selected for further investigations of their potential regulatory networks (Fig. 4a). The Cyan and Plum2 modules showed positive correlation and Blue module had negative correlation. The largest module, Blue, consisted of 2024 genes and thus, the analysis of GO enrichment yielded a long list of significant biological processes (GOs with FDR < 0.001 listed in Additional file 4: File S2b). Among the enriched GOs, biological processes involving skeletogenesis, Wnt signalling pathway and biogenesis of organic compounds were selected for the next analysis step. While almost all of the genes enriched in GOs related to skeletogenesis/Wnt pathway had higher expression in OJA than LPJA, none of their predicted upstream TFs showed jaw-specific differential expression (Fig. 4b, Additional file 4: File S2b). Moreover, an exception within the enriched genes was observed for a paralogue of *prdm1* gene, *prdm1b*, which appeared to have higher expression in LPJA (opposite to the other genes), whereas its paralogue, *prdm1a*, displayed higher OJA expression (Fig. 4b). This might suggest the possibility of sub-functionalisation of *prdm1* paralogues in a jaw-specific and modular manner during cichlid development. We previously found *prdm1a* to exhibit modular gene expression in jaws of these carnivorous Tropheini cichlids as well as other haplochromines cichlids using qPCR [55]

Furthermore, in the Blue module, a long list TFs was predicted upstream of the genes with enriched GOs related to biogenesis of organic compounds. We found *foxq1* and *sox9*, which were among the top 10 predicted TFs, to be differentially expressed between the jaws. Interestingly, the two paralogues of *foxq1* gene (*foxq1a* and *foxq1b*) showed contrasting jaw-specific expression, i.e. *foxq1a* had higher expression in LPJA and *foxq1b* displayed higher expression in OJA, suggesting their modular sub-functionalisation in developing cichlid jaws (Fig. 4b). Most members of the two sub-modules regulated by *foxq1* and *sox9* were highly expressed in OJA with few exceptions such as *tead3a*, *nr2f6a* and *prdm1b*. Interestingly, the *prdm1* paralogues were found to be enriched in all the investigated sub-circuits of Blue module indicating their potential role in linking the effects of organic compounds biogenesis to skeletogenesis in jaws.

The Plum2 module contained 1877 genes. Many of the top 20 enriched GOs for Plum2 module were related to biological processes involving nucleoside metabolism (GOs with FDR < 0.001 listed in Additional file 4: File 2b), therefore we selected these GOs for TF prediction steps. We identified only 2 TFs, *sf1* and *err1/esrra* as upstream regulators of genes enriched in these GOs, and *err1* displayed high expression in LPJA (Fig. 4c). Similarly, most genes in the *err1* related sub-module appeared to have high expression in LPJA (Fig. 4c). We performed the same analysis steps for the Cyan module comprising 1277 genes and these resulted in GOs related to muscle development and myofibril assembly, and *mef2* as top ranked predicted TF (Fig. 4d). Furthermore, we found *mef2a* and most of its downstream genes to be highly expressed in LPJA indicating more active muscle cell differentiation in the surrounding tissues of developing pharyngeal jaw compared to oral jaw (Fig. 4d).

### GO annotation of species-specific modules

The most important species-specific modules in our analysis were the Black and Purple modules containing 2117 and 1358 genes respectively (Fig. 4a) that showed the opposite correlation patterns in the piscivore *C. horei* and the invertivore *G. pfefferi.* The black module, which was positively correlated with *G. pfefferi* has genes annotated for several important pathways (Additional file 1: Figure S8), the most interesting of which was hypertrophy (2.5 enrichment ratio), suggesting an increase in muscle mass in *G. pfefferi*. The other interesting pathways were the androgen receptor pathway (1.39 enrichment ratio) and the ERK/MAPK cascade (1.25 enrichment ratio). The purple module, which was negatively correlated with *G. pfefferi*, but positively correlated with *C. horei* was enriched for the polyol pathway (6.0 enrichment ratio), selenium metabolism (1.9 enrichment ratio), estrogen signalling (enrichment ratio 1.6) and most notably, the delta-notch signalling pathway (1.5 enrichment ratio, Additional file 1: Figure S8).

## Discussion

There is keen interest to identify the genes controlling the craniofacial anatomy of vertebrates in evolutionary biology, particularly those underlying divergent and convergent trophic adaptations. This is especially true for cichlid fishes where the decoupling of the oral and pharyngeal jaw are considered a key innovation driving trophic diversification and adaptive radiation [17]. Jaw morphogenesis, like many ecologically and evolutionarily relevant phenotypes, is a quantitative trait governed by myriad genes. As genes do not function in isolation, understanding gene to gene relationships and their hierarchy is critical to better understand the regulatory pathways underlying adaptive traits. This can be accomplished by linking evolutionary theory with systems biology [56, 57]. GCN analysis is a systems biology approach that transcends single gene level analysis and provides more information about gene–gene relationships underlying complex traits [32]. Here we demonstrate its application in identifying the network of genes underlying a key cichlid ecological adaptation: the functional decoupling of oral and pharyngeal jaw apparatuses. We show that transcriptional modularity of the OJA and LPJA recapitulates their form and functional modularity, exhibiting independently evolving gene expression patterns in the Tropheini cichlids from Lake Tanganyika. Our results suggest that rewiring of GCNs may have facilitated the functional and evolutionary modularity of cichlid jaws. Furthermore, we identify several jaw- or species-specific coexpressed gene modules and incorporate functional annotations to define in silico GRNs governing cichlid jaw morphogenesis.

### Cichlid OJA and LPJA are transcriptionally decoupled and evolving independently

Modularity is a central concept in evolutionary biology as it can enhance ‘evolvability’. The hierarchical clustering of transcriptomes sequenced in this study revealed that gene expression signatures of the OJA and LPJA are conserved across different trophic niches as well as across Lake Tanganyika and Lake Malawi. This suggests that the two jaws have distinct gene regulatory environments, and that this dissociation underlies the key innovation that is their functional decoupling. Based on the divergence time of the cichlid species in this study, this conservation spans at an evolutionary distance of ~ 5.0 MYA (divergence time of Lake Malawi cichlids and the Lake Tanganyika Tropheini) [P. Singh, unpublished data; 58] but this regulatory homology most likely arose as deep as the modification of the gill arches in the craniota. Our findings are consistent with previous studies in mammals showing that tissue-specific clustering of gene expression dominates within major clades, especially in tissues that demarcate a clade [11, 59]. Additionally, in mammals it was also observed that gene expression clustering is consistent with the phylogeny until about ~ 5 – 7 MYA [11]. However, neither the species clustering in the OJA, nor in the LPJA, followed the known phylogeny of these species [58]. We speculate that this lack of consolidation of the gene sequence with gene expression is the result of the recent divergence of these species and jaw adaptations. Analysing transcriptomes from a range of recent and older diverged cichlid species would show if such a consolidation exists along the phylogeny.

The species clustering of LPJA gene expression differed from the OJA suggesting that gene expression, and thus gene regulation of these two units is evolving independently, as would be the case under the model of modularity. This further reinforces the idea that OJA and LPJA are transcriptionally decoupled. As the study species exhibit two highly specialised carnivorous and one omnivorous (i.e. generalist) diet regimes, the differential clustering of the jaws may reflect the unique post-divergence trajectory of each species towards trophic specialisation. In the OJA gene expression clustering, *C. horei* and *L. dardennii* were closer to each other than to *G. pfefferi*, which supports the distinct mouth form and prey catching strategy of the invertebrate picker *G. pfefferi* [60]. Furthermore, in both the OJA and LPJA samples, the Lake Malawi algae grazer *P.* sp. ‘yellow chin Chewere’ clustered closely with the Lake Tanganyika omnivore, *L. dardennii*, and not as sister group to all three Lake Tanganyika endemic Tropheini species, like in the species phylogeny. This is interesting not only because both species largely feed on algae [40] but because Lake Malawi cichlids comprise a related but independent adaptive radiation that is thought be seeded by the same ancestral lineage that seeded the Tropheini in Lake Tanganyika. Instead, the clustering hints at perhaps repeated evolution of gene expression in convergent trophic morphologies.

### Conserved but rewired coexpression networks underlie oral and pharyngeal jaw origin

The jaws of gnathostomes (jawed vertebrates) are ancient structures that evolved from the pharyngeal arches [61, 62]. The number of arches is variable across vertebrates [63] and cichlid fishes belong to the teleost group that have seven pharyngeal arches [64]. The OJ is derived from the first pharyngeal arch, the hyomandibular complex is derived from the second pharyngeal arch, and the most posterior seventh arch forms the PJ [64, 65]. Given that the origin of elements in both the OJ and PJ lies in serially homologous pharyngeal arches, we hypothesised that ancient regulatory networks were modified to yield two novel morphologies, as already demonstrated for the dentition of the two jaws [29, 66–68]. Reorganisation of existing GRNs may have permitted rapid phenotypic evolution in the Tropheini. In particular, modification of *cis*-regulatory modules that govern gene expression within a GRN are known to have significant effect on development and phenotype [27, 69–71]. For example, it was shown that rewiring of GRNs played a role in the body segment modification in insects [72] and the acquisition of an embryonic exoskeleton in sea urchins [73]. Our findings show that the same suite of coexpressed gene modules is deployed in both the OJA and LPJA, suggesting that the gene composition of these modules may have been derived from ancestral pharyngeal arch GRN in jawless vertebrates. Evolutionary rewiring of these ancient GRNs may have first given rise to the OJA from the first pharyngeal arch and subsequently to the pharyngeal jaw from the seventh pharyngeal arch. Furthermore, we show that rewiring manifests not only in the architecture of gene connectivity, but also in the form of differential gene expression and sub-functionalisation of gene duplicates (details discussed below). As rewiring of an ancient gene network has already been suggested for the evolution of teeth on the oral and pharyngeal jaws [29], given that teeth and jaws co-evolved, it is not surprising that similar mechanisms may have driven the evolution of the two jaw structures.

### Jaw-specific co-expression modules

Many members of the Wnt pathway in the jaw-specific Blue module were associated with muscle/skeletal morphogenesis. Cross-talks between Wnt pathway and a dozen other signalling pathways have been described in detail during teleost fish jaw development [30]. As we were not able to identify differentially expressed TFs at upstream of this network, it is likely that one of these interacting pathways influences the transcription of Wnt signal components. In addition, the higher OJA expression of genes like *ap2* (*tfap2*) and *wnt9b* can be assertive evidence for modular specificity of the Blue module, since these genes are determinants of anterior viscerocranium and upper jaw, respectively [74, 75]. Another gene in the Blue module, *hapln1a*, has been shown to be repressed by activated AHR pathway in developing zebrafish jaw [76] and AHR (a major pathway mediating metabolism of cyclic compounds) is among the pathways showing direct cross-talk with Wnt pathway in different zebrafish tissues [77–79]. Additionally, GOs related to organic compounds biosynthesis in the Blue module were highly expressed in oral jaw, and interestingly, paralogues of predicted TFs, *sox9b* and *foxq1b*, are known to be direct downstream targets of activated AHR pathway during zebrafish jaw development [76, 80]. In each sub-module of the Blue module, there were genes with potentially important implications for the divergent morphogenesis of oral versus pharyngeal jaw. For instance, the higher expression *six1b* and *pax3a/b* in *sox9-*related sub-module could indicate the presence of fast-twitch muscle fibres and anterior viscerocranial specific muscle in the surrounding oral jaw tissues [81, 82]. However, the higher expression of *prdm1a* in oral jaw muscles might lead to opposite outcome of decreased fast-twitch muscle fibres [83]. In zebrafish, *prdm1a* is also required for posterior pharyngeal skeletal formation [84], on the contrary, we found increased oral jaw expression of *prdm1a* whereas its paralogue, *prdm1b,* was highly expressed in pharyngeal jaw. This could potentially be an indication for sub-functionalisation of the paralogues as well as potential changes in modular function of *prdm1a* along the anterior–posterior axis in viscerocranial skeleton of cichlids compared to zebrafish. Using qPCR, it was shown that cichlids from all major East African radiations express *prdm1a* modularly and differentially in oral and pharyngeal jaws [55]. It should be noted that *prdm1a/b* were enriched in all the three Blue sub-modules (Fig. 4b). A similar event was observed for *foxq1* paralogues, i.e. *foxq1a* was higher in oral jaw but *foxq1b* showed increased expression in pharyngeal jaw, and again in zebrafish an opposite expression pattern is reported for *foxq1b* along the viscerocranial anterior–posterior axis [80].

The other examples of interesting genes were *lhx6* and *nr2f1* in *sox9*-related Blue sub-module with pivotal role in zebrafish dentition and upper jaw specification, respectively [85, 86] (Fig. 4b). Also, a highly-conserved gene with crucial role in coordination oral jaw development and elongation, *satb2*, was enriched in *foxq1*-related sub-module (Fig. 4b) [87, 88]. *Satb2* delineates a developmental module within oral jaw by controlling the formation of distal elements of both the upper and lower jaws. The later evidence suggests *satb2* as a prime candidate for modular evolvability of vertebrate oral jaw by generating variations in length of the distal elements [89]. The above findings provide first insights into potential transcriptional regulation of these important genes by *sox9* and *foxq1a/b* during oral jaw development in fish.

Genes in the Cyan and Plum2 modules had opposite gene expression direction to genes in the Blue module (i.e. increased expression in LPJA apparatus) (Fig. 4c, d). The TFs, *err1/esrra* and *mef2a*, with increased LPJA expression were predicted at upstream of these modules. *Esrra* is a nuclear receptor regulating estrogen mediated signalling pathway in musculoskeletal system [90, 91]. *Esrra* is required for the formation of mainly posterior viscerocranial skeleton [91], however, its own expression is not induced by activated estrogen pathway during zebrafish viscerocranial development [92]. Markedly, *esrra* is shown to be a potential target of AHR pathway in zebrafish [93], and at the same time it can repress the activity of Wnt pathway during osteoblast differentiation [94]. Therefore, *esrra* seems to be yet another candidate for mediating cross-talk between AHR and Wnt pathways, which appeared to be distinctly activated in oral and pharyngeal jaw apparatus of the cichlids. The enriched GOs for *esrra*-related sub-module were involved in nucleic acid metabolism but little is known about the effects of estrogen signal on nucleic acid metabolism during development, even though such effects are already implicated in adult zebrafish and carp [95, 96]. The enriched GOs for *mef2a*-related sub-module were involved in skeletal muscle formation and *mef2a* is a well-known TF controlling skeletal muscle growth and differentiation [97]. Importantly, *mef2a* participates in adaptive mechanisms by which skeletal myofibers acquire specialised contractile properties (fast- or slow-twitch muscles) [98]. These mechanisms are tightly dependent on the activity of calcium/calmodulin pathway [99] which also plays a role in adaptive morphological changes in the jaw skeleton [100–102].

It should be noted that calcium/calmodulin pathway antagonizes the canonical Wnt pathway in different tissues during vertebrate development [103] whereas it can enhance AHR pathway activity [104, 105]. To this end, we found a GO related to cation/ion transport in the list of enriched GOs for the genes differentially expressed between oral and pharyngeal jaw apparatus. In addition to several genes encoding calcium transport channels (e.g. *cacng6b*, *cacna1sb*, *pkd2* and *atp2b1a*) that are differentially expressed, other genes encoding calcium/calmodulin-dependent protein kinases including *camkk1a/b*, *camk1da*, *camkk2*, and *camk2b1* also show differential expression between oral and pharyngeal jaws. This raises the possibility of differentially activated calcium/calmodulin pathway acting upstream of both AHR and Wnt pathways, which in turn leads to distinct transcriptional pattern of these two pathways between oral and pharyngeal jaws.

### Species-specific coexpression modules

**Fig. 5:**
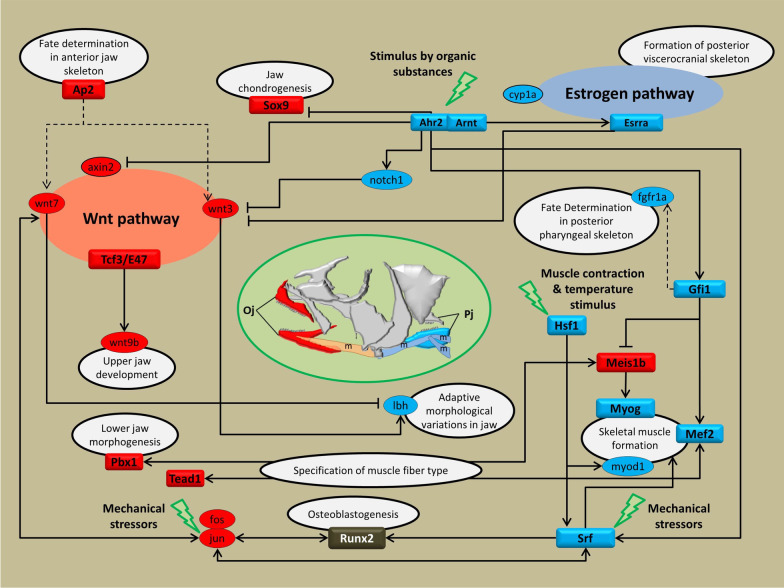
In silico cichlid jaw regulatory networks: highlights of important interactions between in silico annotated gene regulatory networks derived from OJA vs LPJA differentially expressed genes. The in silico annotations and interactions are deduced based on available data for vertebrates in FunCoup 3.0 [51], STRING v10 [52], and a meticulous literature search. The solid lines with arrow and blocked ends indicate potential inductive or repressive regulatory interactions with evidential bases from studies of vertebrates. The dashed lines indicate potential regulatory interaction found in this study but without functional support. The predicted TF(s) upstream of each each sub-module are depicted in square shape gene symbol with their related functions marked. The red, blue and white shadings for genes respectively indicate increased expression in oral and pharyngeal jaws, and no expression difference between the jaws

Interestingly, the Black and Purple coexpression modules had significant but opposite correlations with *C. horei* and *G. pfefferi*. These two sister species are both carnivores but with high specialised and distinct ecomorphologies [60] and diets [40]. *G. pfefferi* has a longer snout that is specialised for picking invertebrates from the soft lake bottom [40]. The Black module that was positively correlated with *G. pfefferi* was enriched for hypertrophy pathways that are known to result in increased muscle mass. Extreme hypertrophied lips have evolved repeatedly in several cichlid radiations to exploit novel food resources [106]. The black module contained *dlx5a* (Additional file 4: File S2b), a transcription factor involved in soft tissue craniofacial morphogenesis that was also differentially expressed in cichlids with nuchal humps [107]. The Black module was also enriched for the ERK/MAPK cascade that plays a key role in the development of the mesoderm, teeth and skeleton and have been shown to be trophic modulators in mice [108]. The MAPK pathway also interacts with the BMP and Wnt pathways, which are both involved in craniofacial development [30]. The Purple module was positively correlated with *C. horei*, a specialised fry-eater with a terminal mouth. The most prominent pathway in this module was the polyol pathway, which is associated with skeletal muscle atrophy [109] and also the Selenium metabolism pathway that has been linked to bone metabolism [110]. Interestingly, the estrogen pathway enriched in the purple module is associated with sex-specific trophic viscerocranial morphogenesis in cichlids [111]. The estrogen pathway interacts with the Ahr pathway (Fig. 5). Finally, the enriched Delta-Notch Pathway in this module is well-established for its role in craniofacial development [112]. Based on our findings, it is likely that the genes in the Blue and Purple modules, play modular roles in determining divergent carnivorous trophic morphologies in the Tropheini.

### In silico GRNs annotated in differentially expressed oral versus pharyngeal jaw genes

The identification of GRNs promises opportunities to unravel the details of complex molecular mechanisms driving phenotypic diversification. Recent efforts have been undertaken to identify GRNs underlying morphological variations of craniofacial skeletal structures in non-model teleost fishes [31, 36, 113, 114]. However, little is known about the details of GRNs determining oral *versus* pharyngeal jaw structures. In this study, we focused on genes that were differentially expressed between OJA and LPJA, and were also enriched for GOs related to musculoskeletal system formation as well as metabolic response to organic compounds. The latter could provide molecular links between mechanisms involved in jaw formation and sensing extrinsic/intrinsic chemical cues, as well as metabolic processes. Based on a thorough literature review and vertebrate databases, we propose an in silico GRN of genes identified in the enriched muscle, skeleton, and response to organic substances biological processes from the differentially expressed genes between OJA and PJA (Fig. 5).

WNT is a major skeletogenesis and bone remodelling pathway [115] and the AHR pathway mediates the effects of environmental chemicals and nutritional compounds during jaw musculoskeletal development through transcriptional regulation of a variety of relevant TFs in Arctic charr [116]. We found distinct activation of components of two major upstream pathways, Wnt and AHR, in oral and pharyngeal jaws, respectively (Fig. 5). Antagonistic cross-talk between the two pathways might be a reason for this discrepancy, for instance, through direct repression of *axin2*, a major TF target of Wnt signal, by activated AHR pathway [79], repression of Wnt by *notch1* after its AHR-dependent induction [117, 118], and/or through AHR-dependent induction of *esrra*, another Wnt pathway repressor (see section below). Genes with modular function in the formation of upper and lower oral jaws, *wnt9b* and *pbx1*, were found to be highly expressed in oral jaw compartment [75, 119], whereas *fgfr1a* determinant of posterior pharyngeal skeleton displayed increased expression in pharyngeal jaw [120]. Finally, we found increased expression of Ap1 complex genes (*fos* and *jun*) in oral jaw which can also mediate the effects of mechanical stressors in jaw [114, 121]. Altogether, these observations represent the complex interaction of genes mainly regulated by AHR and Wnt pathways with the potential to respond to environmental changes that determine distinct musculoskeletal system in cichlid jaws. The in silico GRN presented here provides several candidates for functional and molecular evolutionary studies on cichlid trophic adaptation in the future. An in silico GRN related to skeletal muscle formation displayed increased expression in LPJA apparatus for most of its gene constituents (Fig. 5). This suggests that more active muscle formation occurs in this region, possibly due to higher musculature in the LPJA as this jaw is more active in mastication. In contrast, most downstream members of an identified in silico GRN related to skeletogenesis had increased expression in the OJA, suggesting more active skeletal formation in the oral region. A major mediator of the Wnt signalling pathway during osteogenesis, *tcf3*, and a marker of specific skeletal elements in oral jaw, *tfap2/ap2*, were found to have increased expression in oral jaw [122, 123].

The enriched GO terms with molecular response to stimulus by organic compounds had the highest number of genes and predicted upstream TFs. Of note were components of the Ahr pathway, which can also be activated by diverse endogenous chemicals [124] and thus it perhaps provides a link between exposure to a range of compounds (from metabolic products to environmental toxicants) and jaw skeletogenesis. Remarkably most of the predicted TFs of genes involved in stimulus by organic compounds had oral/pharyngeal jaw differential expression in our analysis and are known to play a variety of roles in skeletogenesis. Two of the TFs in this in silico GRN, *mafaa* and *mafbb*, are of paramount importance because of their responsiveness to presence of extrinsic/intrinsic chemicals and activating signaling cascades required for metabolisms of organic compounds in fish [125]. This has significant implications for understanding the regulatory networks governing adaptive phenotypic plasticity in cichlid jaws [126].

### Limitations of our study

Our study design is correlational and based upon discrete transcriptomes taken at one particular life stage from non-inbred fish reared under standardised conditions. At this stage no information regarding the phenotype – aside of the choice of model species and tissue – were used in constructing the networks. Our analysis is intended to complement those connecting quantitative traits and their genetic basis by providing a wider transcriptomic perspective. The suspensorium of gnathostomes can be traced back in ancestry to the gill arches of the Chordata that show serial homology, which also applies to the apomorphic modifications of the Gnathostomata [63]. The oral jaws originated via modification of the third ceratobranchial, the pharyngeal jaws from modifications of the fifth ceratobranchial arch [17, 62, 65]. Thus, it is possible that sets of conserved ancestral gene networks are acting in all gill arches as well as structures that are derived from it. Another limitation is that we had to restrict the dissection of the pharyngeal jaws to the lower section, which actually represents the ventral portion of the fifth ceratobranchial in terms of structural homology [65]. It would be informative to add transcriptomic data from other less modified gill arches to coexpression network studies in the future to elucidate the set of plesiomorphic conserved genes.

## Conclusion

According to R. A. Raff [127, 128], modularity enhances evolvability by allowing three evolution mechanisms to operate: dissociation (decoupling), co-option (or rewiring), and duplication and divergence. Our study finds that all three of these mechanisms are putatively in play at the transcriptional level in the gene coexpression networks of cichlid jaw apparatuses. We expect that the coexpression modules and in silico jaw regulatory network presented here will be an important resource for futures studies on jaw morphogenesis; especially for those seeking to understand the link between environmental cues and skeletogenesis. Our findings on the OJA are valuable as most cichlids whole-transcriptome studies have focused on pharyngeal jaws. Our findings on the LPJA are also valuable as most vertebrate jaw model systems do not possess pharyngeal jaws. Given the insights gained here, we propose the necessity to embrace a systems biology approach to better understand the complex gene interactions underlying ecologically relevant phenotypes and their evolution.

## Methods

### Study design and sampling

For this study we used three cichlid species (*Gnathochromis pfefferi*, *Ctenochromis horei* and *Limnotilapia dardennii*) from the haplochromine tribe Tropheini in Lake Tanganyika and one haplochromine species from Lake Malawi (*Petrotilapia* sp. ‘yellow chin Chewere’) that were purchased from the aquarium trade or private breeders and reared in the certified aquarium facility at the University of Graz (Austria) (Fig. 1). The fish were raised in standardised aquaria and standardised temperature, light and water conditions, and received the same diet of Spirulina flakes, minimising the effects of plasticity. Once young adult stage was reached, indicated by when mating behaviour was first observed, all individuals were carefully monitored to identify spawning period. Immediately (± 3 h) after the end of spawning, we removed the eggs from the mouth of the females using moderate manual pressure on their cheeks. The eggs were placed in an oxygenated, low speed shaker. Once the eggs hatched and the larvae reached developmental stage 26, which marks the end of postembryonic development as the yolk-sac is absorbed into the body cavity [41], the larvae were euthanized in water containing 0.2 g MS-222/litre and then stored in RNAlater. ﻿By the end of the study the parents of larvae were sacrificed in water containing 0.8 g MS-222/litre. In nature, stage 26 is when the larvae leave the buccal cavity of the mother to begin foraging independently [41]. As we were not focusing explicitly on the developmental pathways, our choice to sample at stage 26 provides a snapshot of growth where the transcriptomes can also provide information on distinct skeletogenesis related processes that are responsive to environmental cues. For each individual larva, the OJA and LPJA were dissected as two separate units and treated as separate samples from here on (Additional file 1: Figure S1). This was an improved design from our previous study where the two jaws were not separated during dissection [42]. The new dissection design here enabled us to study the two units separately and address questions on modularity. The OJA included the upper and lower units of the oral jaw and surrounding soft tissue such as muscle and cartilage that is difficult to remove. The LPJA only included the lower part of the pharyngeal jaw and surrounding soft tissue (muscle, cartilage) and not the upper pharyngeal jaw due to difficulties in dissecting the upper unit. We account for this drawback by avoiding direct comparison of the dorso-ventral patterning of gene expression. Additionally, including bone and soft tissue in the dissection does not violate any biological assumptions about modularity as we know that there is cross-talk between the bone, cartilage, muscle pathways of the trophic apparatus [30]. As we only had one individual of the Lake Malawi *P. sp.* ‘yellow chin Chewere’ species, it was included as the phylogenetic outgroup for the hierarchical clustering analysis in Fig. 2, but excluded from subsequent coexpression and differential expression analyses due to a lack of biological replicates, and because the focus of our study were the Tropheini from Lake Tanganyika (see Fig. 1 for details on the study design and number of biological replicates). Please note that the sampling here was conducted from a different batch of fish and tissues as that in [42] and all new sequencing data was generated for this study.

### RNA library preparation and sequencing

The preserved samples were homogenized by using Lysing Matrix A tubes (MP Bio) in a FastPrep®-24 (MP Bio) homogenizer, further extraction was done using RNeasy Mini Kit (Qiagen). Total RNA qualities and quantities were measured first with NanoPhotometer (Implen) and later with 2200 TapeStation (Agilent) either using High Sensitivity RNA ScreenTapes® or RNA SreenTapes®. Only individuals with a RIN value higher than 7 in one of their extracts were included in this study.

TruSeq® RNA Sample Prep Kit V2 (Illumina) was used to individually indexed paired-end libraries for each sample with unique adapters. At stage 26 in Tropheini species, the pharyngeal jaw is completely ossified and so are mandible, maxilla, and premaxilla of the oral jaw. The articular part of the oral jaw is not ossified, as it is a cartilage (unpublished data from our lab based on histological data from bone and cartilage staining). Thus, the dissection of the OJA (bone and surrounding tissues) of one individual larva produced enough RNA for TrueSeq v2 Kit. We also obtained enough RNA from the lower PJA (bone and surrounding tissues) of one. Therefore, pooling of individuals was not required. Library qualities were checked using D1000 ScreenTapes®, diluted, and 26 libraries were multiplexed to peak molarities and paired-end sequenced on two lanes of Illumina HiSeq® (2 × 125 bp) at the Biomedical Sequencing Facility in Vienna (Austria).

### Differential gene expression analysis

Approximately 11 million paired-end 125 bp sequences were obtained for each sample (315,933,795 PE reads in total; (Additional file 2: File S1). The FastX toolkit (version 0.0.13; http://hannon-lab.cshl.edu/fastx_toolkit/) was used to trim the reads to a length of 92 bp and reads containing 20% or more bases with a Phred Quality score of < 30 were discarded. High quality, filtered reads were mapped to the *Oreochromis niloticus* Broad Institute reference genome [43] using TopHat2 (version 2.1) [44]. Gene expression counts were calculated using HT-seq (version 0.6.1) [45]. Prefiltering was conducted to remove genes with less than 10 total reads across all samples to avoid biases introduced by low coverage genes. Differential gene expression was conducted using DESeq2 [48]. All libraries were normalised simultaneously using default settings recommended by Love et al. [48]. Differential gene expression between OJAand PJA of all species was conducted using the following model: ~ species + jawtype. Wald’s test was used to calculate p.values for the log2-fold change difference. False discovery rate (FDR) [49] correction was used to account for multiple testing (p.adjust value cutoff of < 0.05).

### Gene coexpression network (GCN) analysis

The filtered gene expression table used for differential gene expression analysis was transformed using VarianceStabilizingTransformation implemented in DESeq2 [46], as recommended by the best practices of Weighted Gene Coexpression Network Analysis (WGCNA version 1.68) R-package (version 3.2.1) [32]. As our interest was in the OJA and LPJA comparison, samples from all three species were used as biological replicates for the two jaw units. This provided sufficient sampling power for WGCNA. Hierarchical clustering of samples based on gene expression was conducted to identify sample relationships and outliers. Signed hybrid coexpression networks were constructed using the following steps: (1) Pearson correlation coefficients were used a measure of gene coexpression (2) The Coefficients were raised to a softpower to create an adjacency matrix that was determined with reference to a scale free topology (3) The adjacency matrix is used to calculate the topological overlap distance matrix (4) The topological overlap distance was then used to hierarchically cluster genes (method = average) (5) Groups of coexpressed gene (i.e. modules) were identified using the cutTreeDynamic function with a minimum module size of 30 genes (6) Each module was assigned a colour, and the first principal component (module eigengene) of each module represented the gene expression profile of that module (6) Based on module eigengene (ME) dissimilarity, highly similar modules at a ME distance threshold of 0.25 were merged to obtain the final set of coexpressed gene modules.

Two different sets of coexpression analyses were conducted: (1) Conditional coexpression analysis: OJA and LPJA coexpression networks were separately constructed to investigate the preservation of OJA modules in the PJA network and vice versa. A softpower of 18 was used to construct adjacency matrix (Additional file 1: Figure S2). Module preservation statistics were computed using WGCNA to see how the density and connectivity of modules defined in the reference dataset (i.e. OJA) were preserved in the query dataset (i.e. LPJA) [47]. A permutation test was used to repeatedly permute genes in the query network and Z_score_ was used as a measure of significance. Individual Z scores from all permutations (200) were summarised as a Z_summary_ statistic. (2) Global coexpression analysis: one coexpression network was constructed with both the OJA and LPJA data. A softpower of 6 was used to construct adjacency matrix (Additional file 1: Figure S3). The global jaw modules were correlated with jaw type (i.e. OJA or LPJA) and species in order to identify modules that were jaw-specific and species-specific. In order to do this a gene significance (GS) value based on the Pearson correlation between genes and traits was calculated. Species-specific correlations were coded as follows: *C. horei* vs others, *L. dardennii* vs others*, G. pfefferi* versus others, as recommended for non-binary traits by WGCNA best practices.

### Functional annotation: gene ontology and pathway enrichment analysis

Genes in coexpression network modules and differentially expressed genes were functionally annotated using gene ontology (GO) enrichment analysis and upstream transcriptional regulators/factors (TFs) using WebGestalt, a knowledge-based network enrichment analysis tool for vertebrates [50]. Recent literature on regulatory interactions between the genes of interest, in addition to information available on two comprehensive databases for vertebrate interactome, FunCoup (version 3.0) [51] and STRING (version 10) [52], were utilised to deduce relevant networks of gene interactions. Moreover, the phenotype information of gene mutations was cross-checked with the zebrafish database (ZFIN.org) [53] in order to infer their potential role in jaw skeletogenesis. To do this, we converted gene IDs of the differentially expressed genes to zebrafish orthologues gene IDs with well annotated coding and regulatory sequences (e.g. annotated promoter, introns and 5′/3′-UTR sequences) using the BioMart package [54]. This integrative approach allowed us to in silico annotate enriched genes and pathways within the coexpression modules and differentially expressed genes.

## Supplementary Information

**Additional file 1: Figure S1**. Schematic depiction of the dissection strategy utilised in this study. Red dotted lines mark the cuts made. RNA from oral jaws (upper + lower) and the lower pharyngeal jaw were separately extracted. The dissection included the following tissues: bone, cartilage, teeth, muscle, tendons, fat, and blood vessels. **Figure S2.** Conditional coexpression analysis: fitting scale free topology to establish softpower for constructing the separate OJA and LPJA adjacency matrices. Softpower of 18 was chosen for both. **Figure S3.** Global coexpression analysis: fitting scale free topology to establish softpower for constructing jaw adjacency matrices with OJA and PJA data together. Softpower of 6 was chosen. **Figure S4.** Conditional coexpression analysis: Preservation of modules in the GCNs underlying oral (OJA) and lower pharyngeal jaws (LPJA). **a** Preservation of genes found in LPJA modules in the OJA coexpression network calculated by a Zsummary statistic based on a permutation test that takes into account the connectivity and density of genes in a module. Zsummary < 2 represents lack of preservation (dotted blue line). Zsummary between 2 and 10 implies moderate preservation. Zsummary > 10 supports strong preservation of module. **b** Visual representation of module preservation. Top: LPJA modules in the LPJA GCN. Bottom: LPJA modules in the OJA GCN. **Figure S5.** Global coexpression analysis: Gene co-expression network of the oral and pharyngeal jaws. Dendrograms produced by average linkage hierarchical clustering of 16,669 genes based on topological overlap matrix (TOM). Modules within the network were assigned colours based on the horizontal bar underneath the dendrogram. **Figure S6.** Global coexpression analysis: Barplot of mean trait-based gene significance across modules in the oral and pharyngeal jaw co-expression network. **Figure S7.** Global coexpression analysis: Per module gene significance and connectivity in the oral and pharyngeal jaw co-expression network and connectivity in the oral and pharyngeal jaw coexpression network. **Figure S8.** Global coexpression analysis: enriched pathways in Black and Purple species-specific gene expression modules.

**Additional file 2.** **File S1.** RNAseq mapping read statistics across samples and results from differential gene expression between PJA and PJA.

**Additional file 3.** **File S2. a** Gene ontology enrichment analysis of differentially expressed genes between LPJA and OJA. The enriched genes in GOs related to skeletogenesis, muscle formation and biogenesis of organic compounds were used to predict their potential upstream transcriptional regulators.

**Additional file 4.** **File S2. b** Gene names of genes in jaw- and species-specific modules. Gene ontology enrichment analyses of identified gene networks showing species or jaw specific coexpression. Subsets of related GOs were used to predict upstream transcription factors and downstream sub-module(s) for each network.

## Data Availability

Raw sequence reads are deposited on NCBI SRA (https://www.ncbi.nlm.nih.gov/bioproject/PRJNA626001) and code is available on GitHub (github.com/poojasingh09/cichlid_jaw_modularity_Singh_et_al).

## Abbreviations

OJA: Oral jaw apparatus
LPJA: Lower pharyngeal apparatus
GRNs: Gene regulatory networks
GCN: Gene coexpression networks
TFs: Transcription factors
GS: Gene significance
ME: Module eigengene
GO: Gene Ontology
