# Supplementary material for: Gene coexpression networks reveal molecular interactions underlying cichlid jaw modularity
Source: BMC Ecol Evol. 2021 Apr 22;21:62. doi: 10.1186/s12862-021-01787-9 (PMC8061045; doi:10.1186/s12862-021-01787-9)
Supplement: Supplementary file 1 — Additional file 1: Figure S1. Schematic depiction of the dissection strategy utilised in this study. Red dotted lines mark the cuts made. RNA from oral jaws (upper + lower) and the lower pharyngeal jaw were separately extracted. The dissection included the following tissues: bone, cartilage, teeth, muscle, tendons, fat, and blood vessels. Figure S2. Conditional coexpression analysis: fitting scale free topology to establish softpower for constructing the separate OJA and LPJA adjacency matrices. Softpower of 18 was chosen for both. Figure S3. Global coexpression analysis: fitting scale free topology to establish softpower for constructing jaw adjacency matrices with OJA and PJA data together. Softpower of 6 was chosen. Figure S4. Conditional coexpression analysis: Preservation of modules in the GCNs underlying oral (OJA) and lower pharyngeal jaws (LPJA). a Preservation of genes found in LPJA modules in the OJA coexpression network calculated by a Zsummary statistic based on a permutation test that takes into account the connectivity and density of genes in a module. Zsummary < 2 represents lack of preservation (dotted blue line). Zsummary between 2 and 10 implies moderate preservation. Zsummary > 10 supports strong preservation of module. b Visual representation of module preservation. Top: LPJA modules in the LPJA GCN. Bottom: LPJA modules in the OJA GCN. Figure S5. Global coexpression analysis: Gene co-expression network of the oral and pharyngeal jaws. Dendrograms produced by average linkage hierarchical clustering of 16,669 genes based on topological overlap matrix (TOM). Modules within the network were assigned colours based on the horizontal bar underneath the dendrogram. Figure S6. Global coexpression analysis: Barplot of mean trait-based gene significance across modules in the oral and pharyngeal jaw co-expression network. Figure S7. Global coexpression analysis: Per module gene significance and connectivity in the oral and pharyngeal jaw co-expression netwo [file 12862_2021_1787_MOESM1_ESM.pdf]

Supplementary Figures

Figure S1

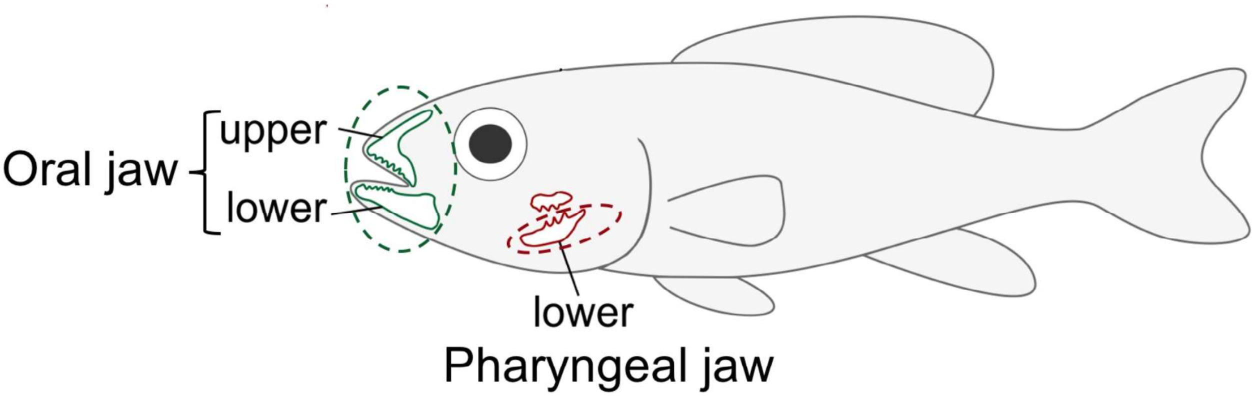

Supplementary Figure 1 Schematic depiction of the dissection strategy utilised in this study. Red dotted lines mark the oral jaw dissection and green dotted lines mark the pharyngeal jaw dissections. RNA was separately extracted from oral jaws (upper+lower) and the pharyngeal jaw (only lower). The dissections included the following tissues: bone, cartilage, teeth, muscle, tendons, fat, and blood vessels.

Figure S2

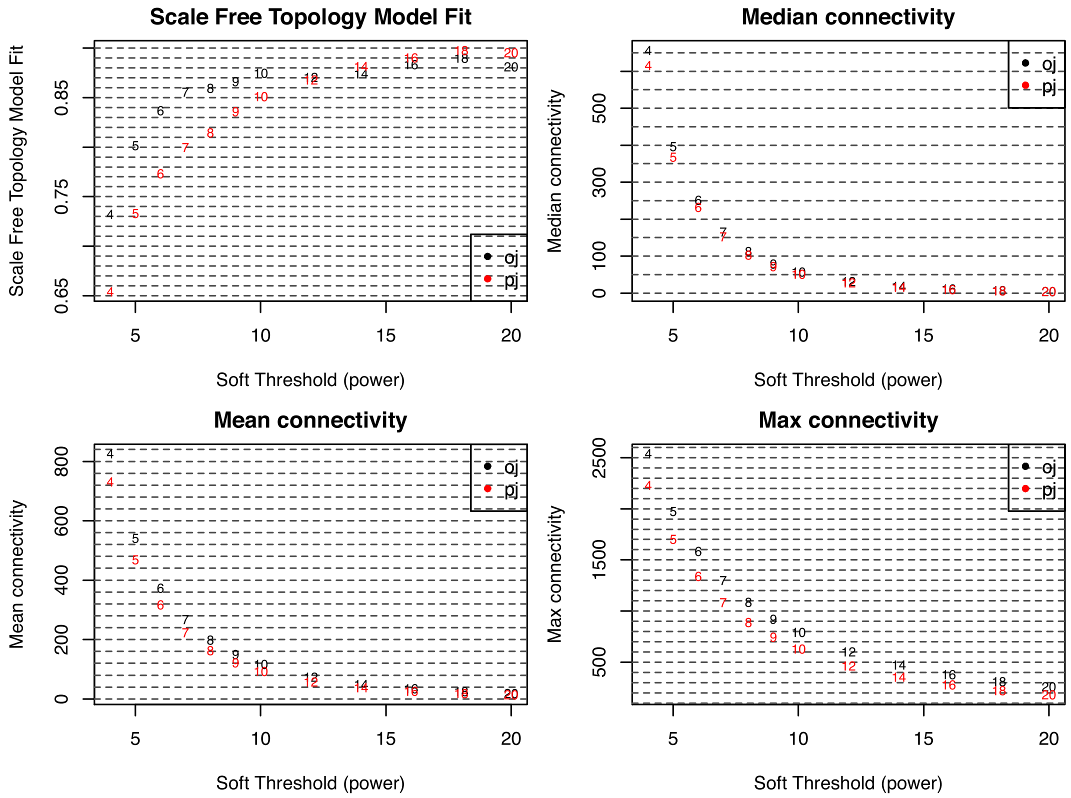

Supplementary Figure 2 Conditional coexpression analysis: fitting scale free topology to establish softpower for constructing the separate OJA and LPJA adjacency matrices. Softpower of 18 was chosen for both.

Figure S3

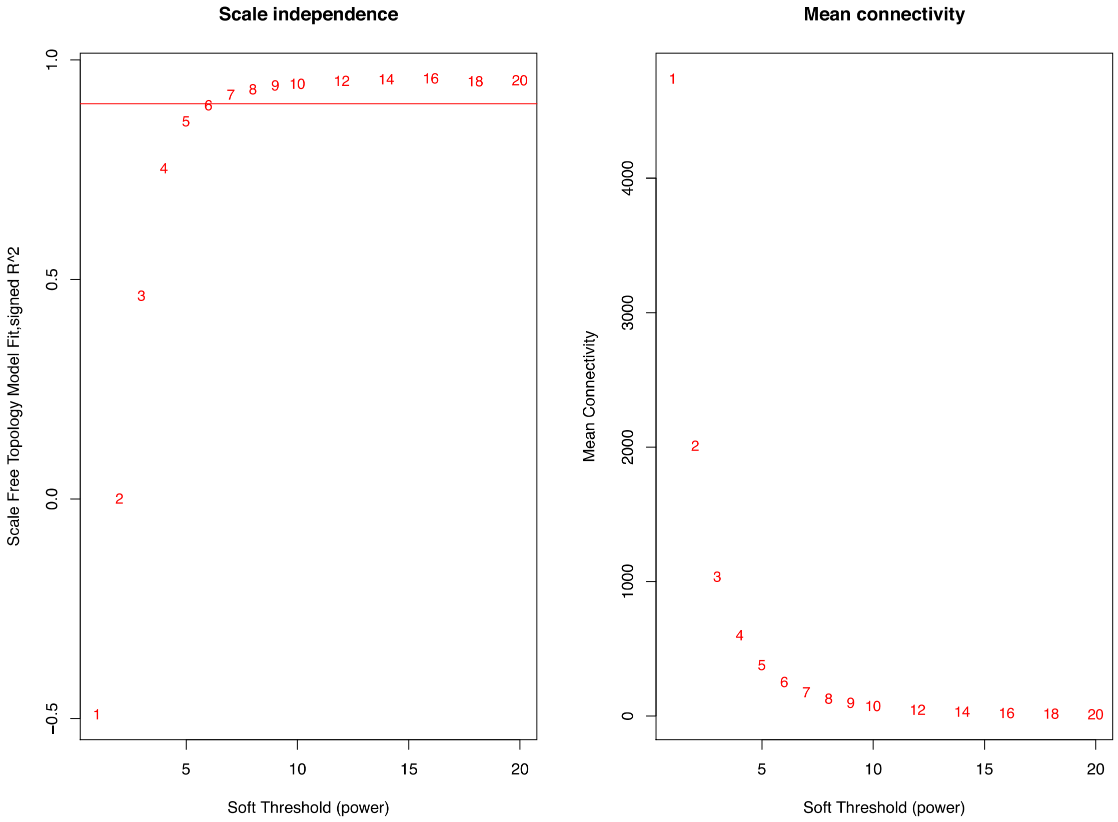

Supplementary Figure 3 Global coexpression analysis: fitting scale free topology to establish softpower for constructing jaw adjacency matrices with OJA and PJA data together. Softpower of 6 was chosen.

Figure S4

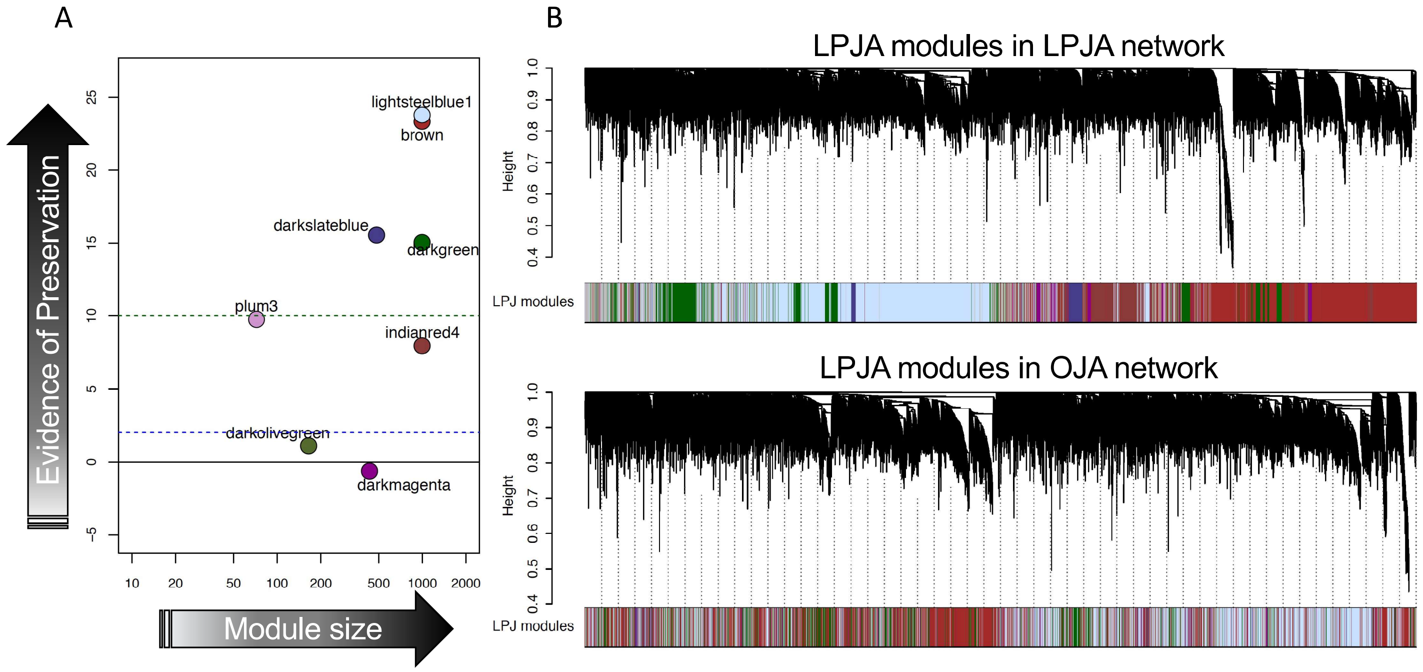

Supplementary Figure 4 Conditional coexpression analysis: Preservation of modules in the gene co-expression networks underlying oral (OJA) and lower pharyngeal jaws (LPJA). (A) Preservation of genes found in LPJA modules in the OJA co-expression network calculated by a Zsummary statistic based on a permutation test that takes into account the connectivity and density of genes in a module. Zsummary < 2 represents lack of preservation (dotted blue line). Zsummary between 2 and 10 implies moderate preservation. Zsummary > 10 supports strong preservation of module. (B) Visual representation of module preservation. Top: LPJA modules in the LPJA gene co-expression network. Bottom: LPJA modules in the OJA gene co-expression network.

Figure S5

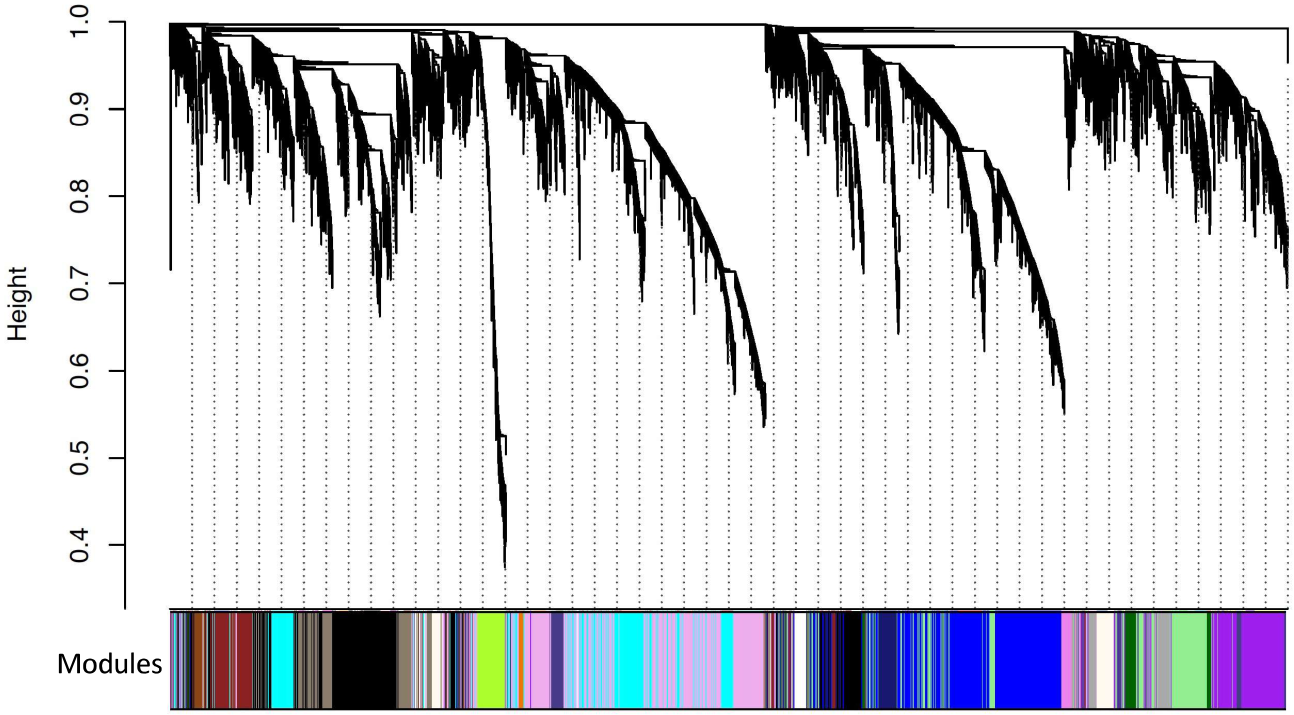

Supplementary Figure 5 Global coexpression analysis: Gene co-expression network of the oral and pharyngeal jaws. Dendrograms produced by average linkage hierarchical clustering of 16,669 genes based on topological overlap matrix (TOM). Modules within the network were assigned colours based on the horizontal bar underneath the dendrogram.

Figure S6

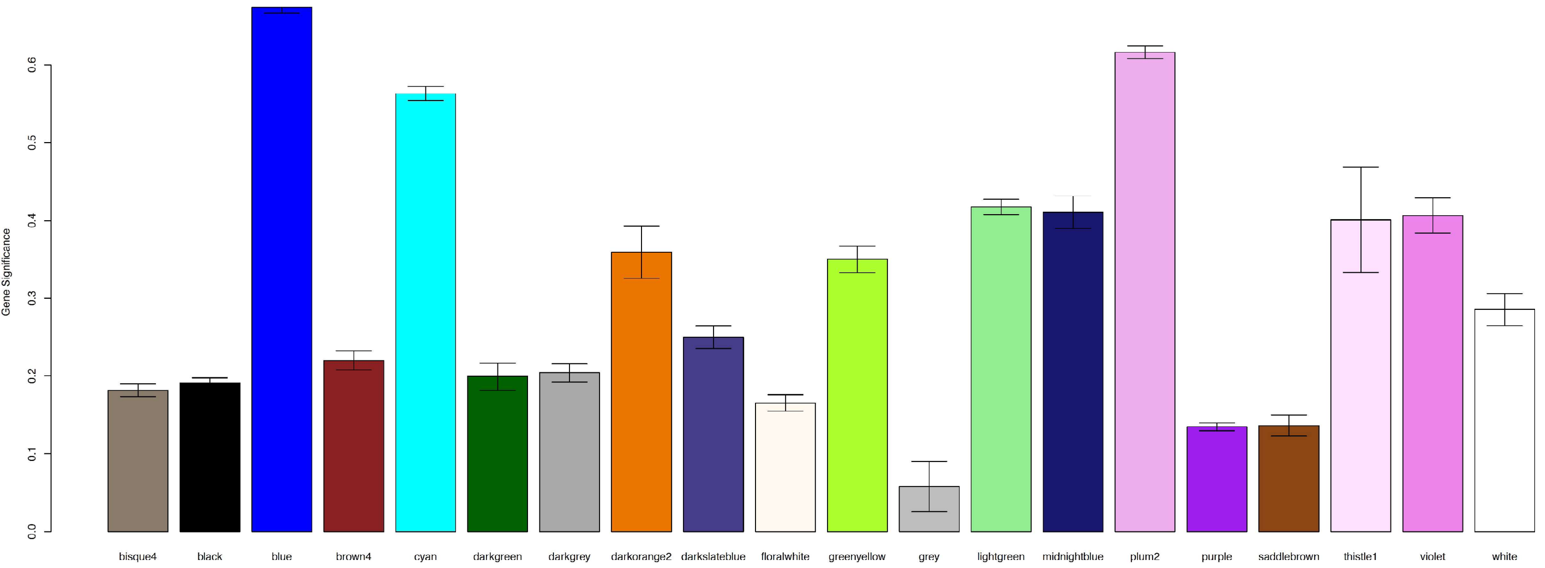

Supplementary Figure 6 Global coexpression analysis: Barplot of mean trait based gene significance across modules in the oral and pharyngeal jaw co-expression network

Figure S7

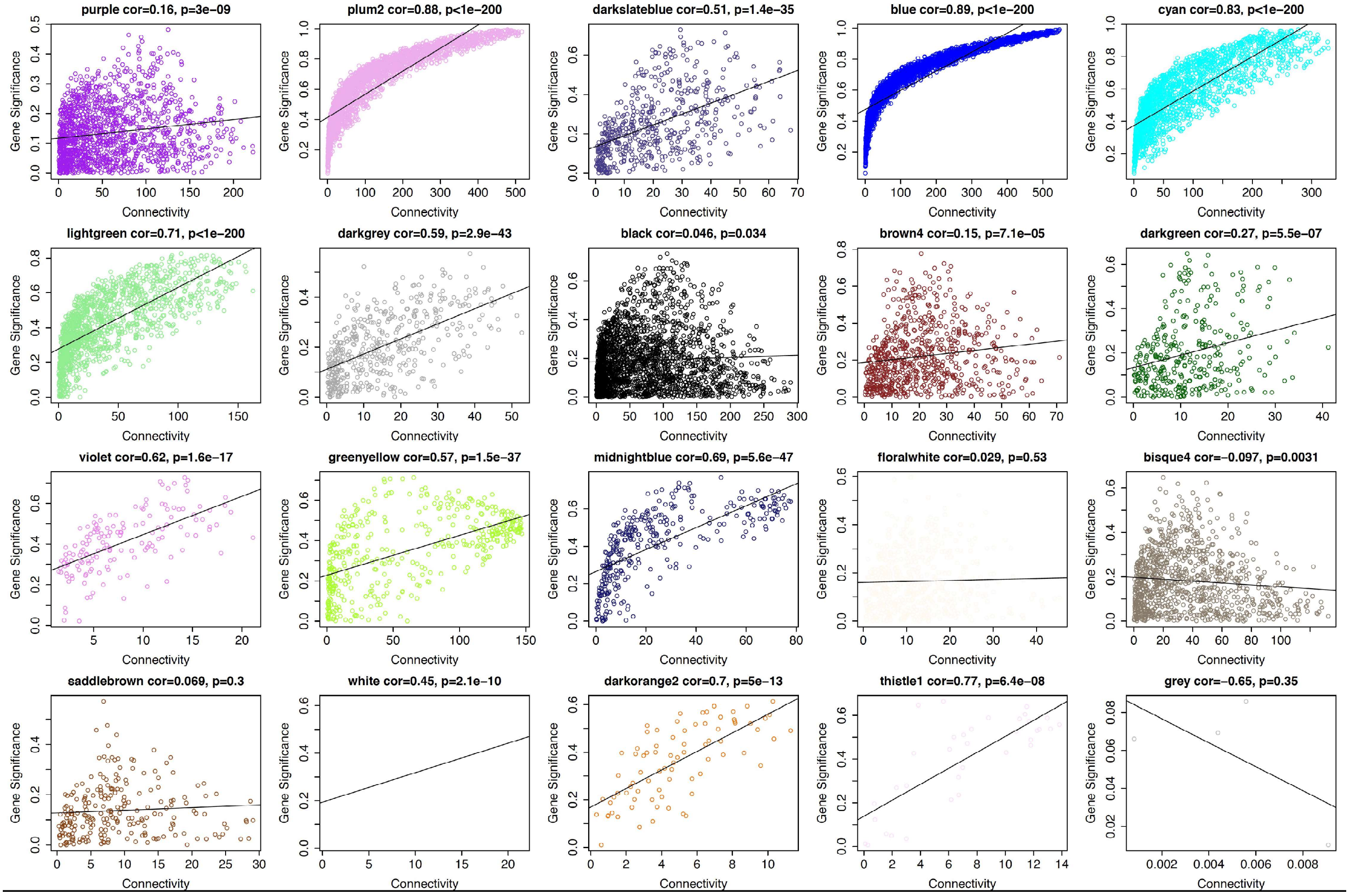

Supplementary Figure 7 Global coexpression analysis: Per module gene significance and connectivity in the oral and pharyngeal jaw co-expression network

Figure S8

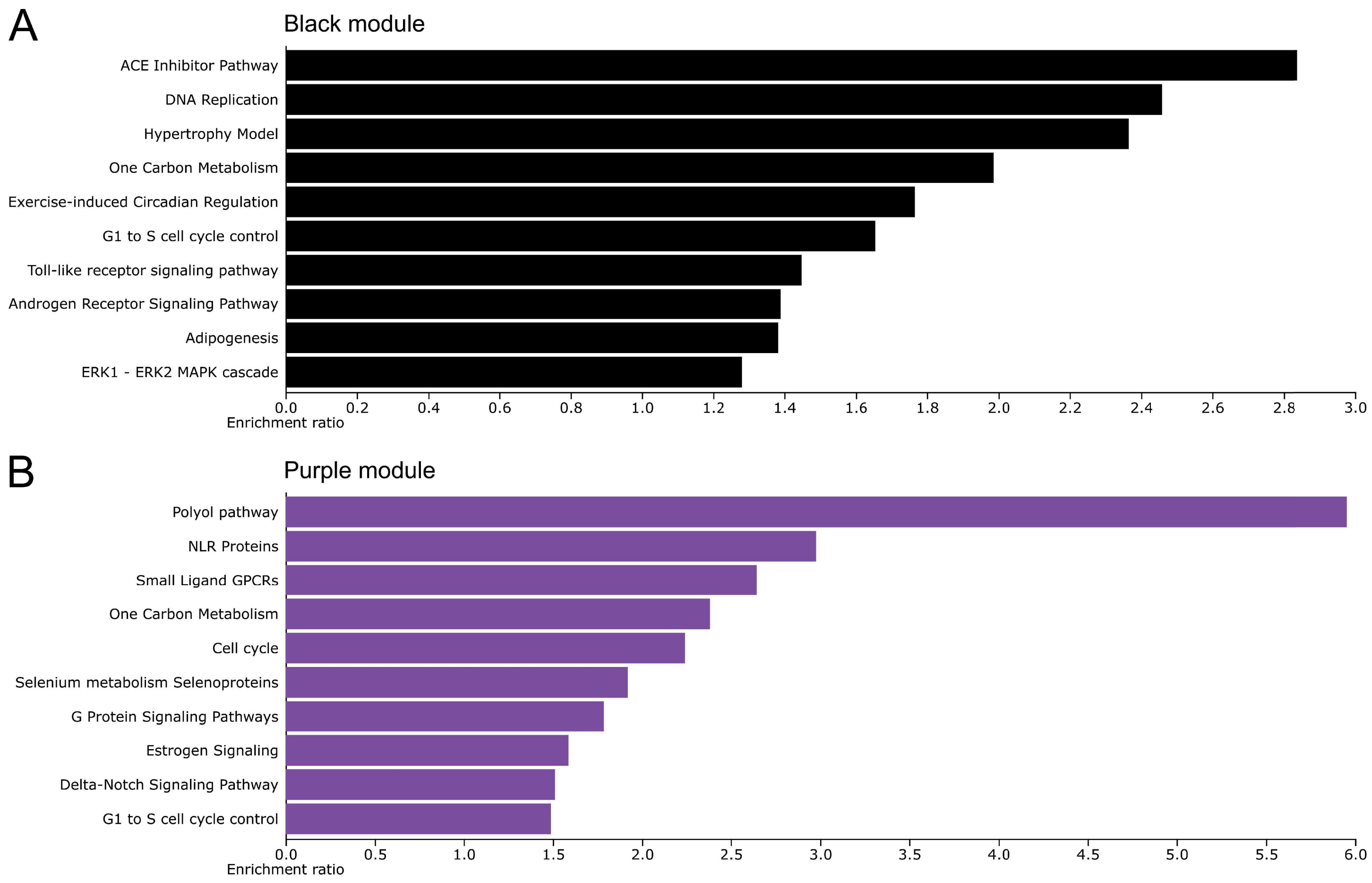

Supplementary Figure 8. Global coexpression analysis: enriched pathways in Black and Purple species-specific gene expression modules.
